# Describing the evidence-base for research engagement by health care providers and health care organisations: a scoping review

**DOI:** 10.1186/s12913-022-08887-2

**Published:** 2023-01-24

**Authors:** Sze Lin Yoong, Katarzyna Bolsewicz, Kathryn Reilly, Christopher Williams, Luke Wolfenden, Alice Grady, Melanie Kingsland, Meghan Finch, John Wiggers

**Affiliations:** 1 Global Centre for Preventive Health and Nutrition, Institute for Health Transformation, School of Health and Social Development, Faculty of Health, Burwood, VIC 3125 Australia; 2 Hunter New England Population Health, Wallsend, NSW 2287 Australia; 3 School of Medicine and Public Health, The University of Newcastle, Callaghan, NSW 2308 Australia; 4 Hunter Medical Research Institute, Newcastle, NSW 2300 Australia; 5 Priority Research Centre for Health Behaviour, The University of Newcastle, Callaghan, NSW 2308 Australia; 6 National Centre for Immunisation Research and Surveillance, Sydney Children’s Hospital Network, Sydney, NSW 2145 Australia

**Keywords:** Research engagement, Research capacity building, Health care providers

## Abstract

**Background:**

Having a research-engaged health and medical workforce is associated with improvements in clinical outcomes for patients. As such, there has been significant government investment internationally to support health care organisations and services to increase staff engagement with research.

**Objectives:**

This scoping review sought to provide an overview of the literature describing strategies employed to increase research engagement by health care providers and organisations, and to undertake a qualitative analysis to generate a list of research engagement strategies.

**Methods:**

A scoping review using systematic search strategies was undertaken to locate peer-review publications and grey literature related to research engagement by health care providers and organisations. Research engagement was defined as a ‘deliberate set of intellectual and practical activities undertaken by health care staff and organisations to conduct research’. A database search of electronic records was performed with no limit on publication date. Publications were included regardless of study type (excluding systematic reviews) and categorised as either databased (presenting data or new analysis of existing data) and non-databased (no new data or analyses). Databased publications were further classified according to study type, study design and setting. A qualitative synthesis using a Framework Approach was undertaken with all studies that described a strategy to improve research engagement.

**Results:**

A total of 152 publications were included in this study with 54% categorised as non-databased. Of the databased articles, the majority (72%) were descriptive studies describing prevalence of correlates of research engagement, 17 (25%) described intervention studies where only two were controlled studies. The following research engagement strategies were identified: i) dual skilled team/staff, ii) resources or physical infrastructure, iii) incentives, iv) leadership support of research, v) education/training, vi) networks, vii) forming partnerships or collaborations and viii) overall leadership structure of entity.

**Conclusions:**

The literature on research engagement is primarily opinion-based and descriptive in nature. To provide the evidence needed to inform strategies, this needs to progress beyond descriptive to more rigorous well-designed intervention research.

**Supplementary Information:**

The online version contains supplementary material available at 10.1186/s12913-022-08887-2.

## Introduction

Health services are under continuing pressure to improve the quality of care they provide and the outcomes of such care for patients. To address this, health services employ a wide range of strategies such as the implementation of new treatments, devices and models of care, the recruitment of skilled staff, professional development of existing staff, investment in quality improvement staff and initiatives, development of IT and other care delivery support systems, tools and resources, and establishment of care delivery and performance monitoring systems [1–5].

The conduct of research in health services, or research engagement by health service providers, has also been suggested to have additional benefits to the quality of patient care and outcomes through indirectly modifying the culture and practice of care delivery [6, 7]. Research engagement has been variably defined with one review describing clinician research engagement as a ‘deliberate set of intellectual and practical activities undertaken by health care staff and organisations to conduct research’ [8]. Systematic reviews have reported improvements in health outcomes and provision of care where clinicians and organisations are research-engaged [6, 7]. Further, several longitudinal studies have reported that provider and/or health care organisation participation in clinical trials can result in lower mortality rates and greater adherence to clinical guidelines [9]. This greater focus on ‘knowledge production/generation’ by health service end users, and engagement of such end users in identifying research priorities has been suggested to promote better quality health care and patient outcomes [10]. In response, a shift in health and medical research and health service delivery policies toward increasing the engagement of health care services in the conduct of research is occurring internationally [11–13].

One example of this is  the National Institute for Health Research (NIHR) in England where 10 million pounds was invested to establish nine collaboration units (Collaborations for Leadership in Applied Health Research and Care (CLAHRCs)) in 2008 aimed at engaging NHS staff to participate in research to improve patient outcomes [11, 12]. A number of funding structures have been put in place to support the integrated Knowledge Translation (iKT) research model in Canadian hospitals to increase researcher’s collaboration with ‘knowledge users’ (i.e. health care providers/users) to increase the generation of evidence that is more readily able to be implemented into practice [14]. In Australia, over $10 million was invested in building Advanced Health Research and Translation Centres (AHRTC) and Centres for Innovation in Regional Health (CIRHs) to facilitate collaboration between researchers, health care and industry stakeholders to undertake applied research directly relevant to improve patient care [13].

### Identifying the research question

 Although healthcare professionals, policy makers and the public at large recognise that research engagement by health care organisations is worthwhile, little is known about the evidence base surrounding research engagement and the type of strategies that have been used to facilitate increased research engagement. Previous reviews examining this have examined research engagement together with how to increase clinicians’ application of research in practice (i.e. implementation science). A review by Boaz et al. examined potential mechanisms between clinician engagement with research and improved health service and patient outcomes [6]. The review found that supporting clinicians to undertake research was a distinct behaviour from increasing clinicians’ implementation and multifaceted strategies are required to support research engagement, findings consistent with other reviews describing research co-production. For example, a review by Gagliardi et al. provides a summary of how integrated knowledge translation approaches have been applied to target decision makers’ engagement with research and described a range of individual and structural barriers and enablers that need to be targeted to support research engagement by health care organisations [15]. The previous reviews examining clinician research engagement specifically however have focused on describing the impact of capacity building strategies that target individual factors only [16, 17]. Our review seeks to extend on this literature by providing an overview of the different strategies that can be used to promote research engagement by health care providers [16]. In areas such as this where terminology, types of strategies and outcomes are unclear, an examination of the volume and characteristics of available research is needed to provide an overview of the evidence available to inform practice and policy making, and to assist in identifying gaps and areas for future research investment to increase health provider/ organisation research engagement [18]. Scoping reviews are recommended to provide an opportunity to clarify key concepts; describe gaps in the research; and characterise the types and sources of evidence to inform practice, policymaking and future research [18].

## Objectives

As such, to identify gaps in the area and opportunities for further research, this study aimed to: i) describe the percentage, type and study design of publications that examined research engagement by health care providers and organisations and ii)provide a list of the types of strategies used to promote research engagement by health care professionals and organisations reported in the literature.

## Methods

### Study design

A scoping review using systematic search processes was undertaken to locate peer-reviewed publications and grey literature related to ‘research engagement by health care providers and organisations’. The manuscript was reported consistent with the PRISMA-ScR [19]. No protocol was registered; however a copy can be provided from the corresponding author upon request. The definition of research engagement applied in this study was informed by a comprehensive commissioned review undertaken by Hanney et al. (2013) which distinguishes between ‘engagement with research’ and ‘engagement in research’ with the former including a less substantial involvement at an organisational level and relating more to receiving and transmitting research findings, more conventionally known as knowledge translation or implementation (evidence-based practice) in the literature [8].

Similar to the review by Hanney et al., this study focused on ‘engagement in research’, defined as a ‘deliberate set of intellectual and practical activities undertaken by health care staff and organisations to conduct research’ [8]. This definition requires health care providers, services and organisations to have a more involved role in the entire research process including in its design, commissioning, conduct and dissemination [8].

This review applied methods consistent with the Cochrane Handbook for conduct of scoping reviews, and was based on the updated framework outlined by Peters et al. [20, 21]. This involved; i) identifying the research question; ii) identifying relevant studies; iii) study selection; iv) charting the data; and v) collating, summarizing and reporting results.

Systematic search processes and broad eligibility criteria were developed by the research team apriori, however consistent with recommendations for the conduct of scoping reviews, the search was supplemented with additional articles based on advice/recommendations from policy makers and experts in the field known to the research team, as well as additional grey literature searches using search terms identified from the initial electronic search (see Additional file 1).

### Identifying relevant studies

A database search of electronic records was performed in the following electronic databases with no limit on date, MEDLINE (1950-); EMBASE (1947 -)’ PsycINFO (1950-); Academic Search Ultimate; CINAHL on the 27^th^ December 2019.

As research engagement is not homogenously defined in the literature and consequently not always clearly indexed in electronic databases, the search strategy was developed based on extensive consultations and piloting of search terms with a university health systems librarian. The librarian, acting in line with Peer Review of Electronic Search Strategies (PRESS) 2015 Guidelines, assessed all aspects of the search terms, subject headings, search strings, limits and filters to ensure they were conceptually and functionally accurate in relation to the research question [22]. This strategy was adapted from that previously employed in a comprehensive review examining the association between research participation/engagement and health outcomes and refined to suit the purposes of this review [8]. The search strategy was kept intentionally broad, consisted of search terms for research engagement combined with search terms for health care providers/organisations. The reference list of all included studies were screened to identify further studies that could be relevant. Subsequently, a search of the grey literature using Google was undertaken using key terms identified from articles included from the electronic database search. Five separate searches were undertaken and included entering ‘research capacity building, collaborative activities/research, health research capacity strengthening and research engagement’ into the Google Scholar search engine and reviewing the first 100 hits in June 2019. Four policy makers and research experts provided additional articles that were relevant to the review aims.

### Study selection

#### Inclusion and exclusion criteria

The main concepts of the review question which shaped the inclusion and exclusion criteria for the search can be found in Table 1.

**Table 1:** PCC (population, concept, context) elements of the review question

PCC Element	Criteria
P (population)	Healthcare providers/organisations. Health care providers are defined as those who study, diagnose, treat and prevent human illness, injury and other impairments in accordance with the needs of the population they serve. Health care organisation as the setting in which healthcare providers provide these services
C (concept)	Research engagement by healthcare providers or organisations, where research engagement is described as a ‘deliberate set of intellectual and practical activities undertaken by health care staff and organisations to conduct research’ [8]. Research engagement strategies are defined as activities or actions taken to increase research activities and leadership by health care providers and organisations’
C (context)	Language limit: English Types of studies: Peer-reviewed and grey literature, and all types of study design (excluding systematic reviews) and no limit by country

Studies that examined research engagement were included regardless of study type. Articles not published in English were excluded from the study. Studies that examined appraising or applying the evidence solely without actual conduct of research, those that examined ‘how’ to move research into practice (i.e., implementation science) and dissemination of research findings exclusively, as well as those undertaken in non-health care settings/or with non-health care practitioners were excluded.

#### Screening

The search, screening and data charting processes were systematic and undertaken by two independent authors. Abstract and full text screening was undertaken independently by two authors (SY/KB/MF/AG/MK) using COVIDENCE [23]. Where there was disagreement, a third reviewer resolved any differences (LW). All studies regardless of study type were included as long as they explored research engagement (defined above).

### Charting the data

Charting data from included publications was conducted according to the data classifications outlined below and consistent with previous studies [24–26].

A standardised data charting tool (see Additional file 2) was developed to chart the following additional information: author, year of publication, country where research was conducted (or first author affiliation if review), study design, study type, study setting and the detail of the intervention (verbatim from the text). A detailed instruction sheet with how to code each study was developed to ensure standardised data charting. All extraction were undertaken by two authors in Microsoft Excel, and all differences resolved via discussion (KB/SY/JW/CW).

#### Publication classification

All studies were categorised as either databased (i.e. data-driven, presenting data or new analysis of existing data relating to research engagement) or non-databased (i.e. not data-driven, no data or new analyses), as a means of differentiating between the different levels of evidence [27]. Databased publications were further classified according to study type, study design, translation levels and study setting.

*Study type:* Databased studies were further classified into quantitative or qualitative based on that reported by the authors. Where this was not explicitly described, we based this on the type of analysis used. Studies that used both quantitative and qualitative methods were classified as mixed methods. Databased studies were also categorised into measurement, descriptive or intervention research consistent with definitions from previous studies [24–28]. The following definitions were used:

1. **Measurement:** Papers developed or examined the qualities of a measurement instrument such as reliability, validity, or acceptability. Data collection methods included the use of questionnaires, interviews, physiological assessments, risk screening and observations. Papers that focused on both measurement and descriptive issues were coded as measurement research.
2. **Descriptive:** Papers exploring the frequency, patterns, correlates or predictors of research engagement strategies. These include epidemiological studies examining frequency or patterns of risk factors and correlates of research engagement.
3. **Intervention:** Papers that tested the effectiveness of an intervention to increase research engagement and/or providers. Intervention publications will be defined by the research aims rather than the study design or type of intervention. Papers that focused on both descriptive and intervention issues will be classified as intervention research.

Non-databased articles were categorised as below consistent with previous studies [27]:

a **Discussion papers or commentaries:** Editorials, comments, letters, news or interviews. These articles did not present original data or describe a specific research project or intervention.
b **Case reports:** Articles in which the publication description indicated that it was a case report.
c **Program description:** Descriptions of methods or processes undertaken for research engagement. This category included articles that described an intervention or health initiative being applied, or that had the potential to be applied, but in which no data-based evaluation was reported.

*Study design:* Those with an intervention focus were further classified according to the levels of evidence (including cross-sectional, case control, cohort studies, non-randomised controlled trial, quasi randomised controlled trial, and randomised controlled trial) [29].

*Study setting:* The study setting was recorded as either hospital(s) or a department within hospitals, community health settings, primary care, health care setting more broadly (not specific), and networks or collaborations at a national or international level or other.

#### Qualitative synthesis

To produce a list of strategies used to promote research engagement (i.e. research engagement strategies), we undertook a qualitative synthesis of data from databased intervention studies, and all non-databased studies, using a Framework Approach (FA) [30, 31]. This was undertaken on the narrative description of the research engagement strategies that were extracted verbatim from the relevant studies. The FA is a type of systematic, transparent qualitative content analysis composed of several inter-related stages that allow for a structured case- or theme-based analysis of data [30, 31]. FA is conducted by a team, where analysis is aided by collaborative development of highly structured outputs (charts/displays) providing an accessible visual reference that can be interpreted by people with little qualitative research experience [31]. Such qualitative methods of synthesising review findings have been recommended to generate broad findings/key themes and to highlight opportunities to advance the evidence-base [32, 33].

As part of this, the team (JW, KB, SY, CW) employed five stages of the analysis, including; 1) familiarisation with the data; 2) identification of recurrent and important themes; 3) coding; 4) grouping codes into an analytical framework; and 5) applying an analytical framework to data [31]. The entire process was facilitated by a skilled qualitative researcher (KB). The focus of the analysis was to produce descriptive accounts to answer predefined research questions (elements of research engagement strategies that are intended to, have been used to, or have been evaluated in the context of improving research engagement by health care providers and organisations). The analytical framework(s) emerging from the qualitative data analysis was intended to be pragmatic in nature, tailored to produce information that can be used to inform public health practice. Aligned with the project specifications and aims, we applied a positivist approach to qualitative synthesis, where we comprehensively searched for empirical findings and then accumulated and described these findings [32].

## Results

### Collating, summarising and reporting results of included studies

**Fig. 1:**
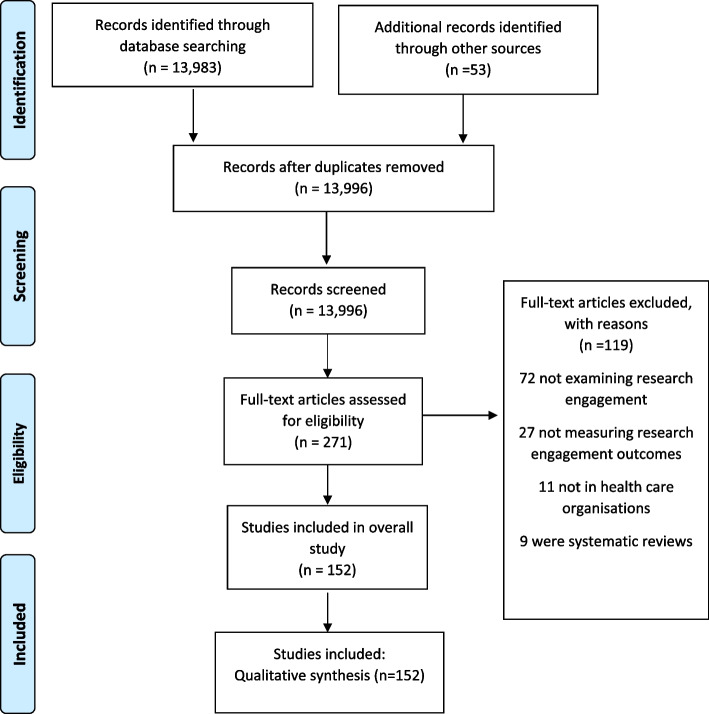
Flowchart outlining number of studies included at each review stage

From the electronic search, 13,983 references were screened by two reviewers and 53 were identified through other sources. A total of 13,996 articles were screened after removal of duplications. A total of 271 articles were included in the full text screen and 152 were included in this study (see Fig. 1 for PRISMA diagram). This total included 107 from the database search, 40 searching from the grey literature included articles and and five based on expert recommendations, resulting in a total of 152 records for inclusion (see Fig. 1).

A range of terminology was used to describe clinician and health care organisation research engagement including building research capacity, collaborative research, academic clinicians, building research infrastructure in health care, research participation, translational research, fostering clinician led research, exchanging knowledge, research networks, practice-research collaboration, integrated knowledge translation, research-based practice and research co-production.

Most studies were conducted in or had a first author located in the United States (*n* = 46), Australia (*n* = 41), the United Kingdom (including England, Ireland and Scotland, *n* = 36) and Canada (*n* = 17). The remaining were in New Zealand (*n* = 2), Norway (*n* = 1), Denmark (*n* = 1), the Netherlands (*n* = 1), Sweden (*n* = 1), Spain (*n* = 1), Switzerland (*n* = 1), France (*n* = 1), Ireland (*n* = 1), Japan (*n* = 1), and two in low to middle income countries.

### Publication classifications

Of the 152 included records, 68 (44%) presented new, synthesised data (databased) and the remaining 84 (56%) were non-databased. Twenty-four studies were published before the year 2000, 46 were published between 2000 and 2009 and the remaining 82 were published after 2009 (2010–2019) (see Table 2).

i. **Study type**

**Table 2:** Study characteristics for all included studies *two studies are missing from this table, apologies for this and need to be added in these - are Norman 1987 and cooke 2005

Author name, Year	Country	Quantitative/Qualitative	Study type	Study design	Setting and participants
Abbot 2005 [34]	England	Qualitative	Intervention	Cross-sectional	Primary care practices, practice nurses
Akerjordet 2012 [35]	Norway	Quantitative	Descriptive	Cross-sectional	University hospital, nurse managers
Albert 2015 [36]	USA	NA	NA	Program description	Midwest healthcare system, nurses
Albert & Siedlecki 2008 [37]	United States	Qualitative	Descriptive	Cross-sectional	
Alcock 1989 [38]	Canada	Qualitative	NA	Commentary	Clinical
Armstrong 2009 [39]	United States	NA	NA	Commentary	Clinical
Aronson 2011 [40]	England	NA	NA	Commentary	None specific
Axon 2012 [41]	United States	Quantitative	Descriptive	Cross-sectional	Hospital, hospital medicine researchers
Babl 2006 [42]	Australia New Zealand	NA	NA	Commentary	Network, Paediatric Emergency Research
Bacigalupo 2006 [43]	England	Quantitative	Descriptive	Cross-sectional	Primary care trust, general practice staff, PCT employed staff, social care staff
Bailey 2006 [44]	Australia	NA	NA	Commentary	Community, Aboriginal health workers
Bakken 2009 [45]	United States	Mixed Methods	Descriptive	Cross-sectional	Ambulatory Care Network Sites, community clinicians
Balakas 2011 [46]	United States	NA	NA	Commentary	Hospital, nurses
Barnsteiner 2010 [47]	United States	NA	NA	Commentary	Hospital, nurses
Beeson 2014 [48]	United States	Quantitative	Descriptive	Cross-sectional	Community health centres, community clinicians
Berger 2015 [49]	United States	NA	NA	Program description	Healthcare system, nurses
Black 2016 [50]	Canada	NA	NA	Program description	Healthcare organisation, nurses and allied health professionals
Blevins 2010 [51]	United States	Mixed methods	Descriptive	Cross-sectional	Clinical Partnership Program at the Veterans Healthcare Administration
Bogo 1992 [52]	Canada	NA		Program description	Healthcare settings and university, social work faculty and healthcare staff
Boydell 2012 [53]	Canada	Qualitative	Descriptive	Cross-sectional	Paediatric care settings, physician researchers working in paediatrics
Bragg 2011 [54]	United States	Quantitative	Intervention	Repeat cross sectional	Department of Veterans Affairs (VA), staff within the centres/participating in the program broadly
Brauer 2007 [55]	Australia	NA	NA	Commentary	Physiotherapy Rehab Network, physiotherapists
Brewer 2009 [56]	United States	NA	NA	Program description	Tertiary care hospital, nursing staff
Brown 1994 [57]	Canada	NA	NA	Commentary	Clinical more broadly, occupational therapy
Bryar 2003 [58]	United Kingdom	Qualitative	Descriptive	Cross-sectional	Primary care, nurses, midwives, GPs, practice managers, health promotion specialists
Bullock 2012 [59]	England	Qualitative	Intervention	Cross-sectional	Healthcare organisation, healthcare managers
Bullock 2013 [60]	England	Qualitative	Intervention	Post intervention assessment	
Callard 2012 [61]	England	NA	NA	Commentary	Healthcare broadly, none specific
Castonguay 2011 [62]	United States	NA	NA	Program description	University counselling centres, counsellors
Castonguay 2015 [63]	United States	NA	NA	Program description	Psychology training clinic, none specific
Chan 2010 [64]	Australia	NA	NA	Program description	Clinical setting, nurse researchers
Chapman 2005 [65]	Australia	Quantitative	Intervention	Pre-post non-controlled study	Emergency department, health campus, nursing staff
Chassie 1990 [66]	United States	NA	NA	Program description	Hospital, nursing staff
Chester 2007 [67]	United States	NA	NA	Commentary	None specific, nursing staff
Cleary 2010 [68]	Australia	NA	NA	Commentary	Mental health settings more broadly, none specific
Cluver 2014 [69]	United States	Quantitative	Intervention	Prospective	Psychiatric setting more broadly, medical students
Cole 2014 [70]	Canada	Qualitative	Intervention	Retrospective reviews	Healthcare setting more broadly, none specific
Cooke 2005 [71]	England	NA	NA	Cross-sectional	Research development support unit
Cooke 2014 [72]	England	NA	NA	Program description	CLARHC South Yorkshire, none specific
Cooke 2015 [73]	England	Quantitative	Descriptive	Program description	CLARHC North of England
Corchon 2011 [74]	Spain	Quantitative	Intervention	Quasi-experimental	Hospital, nurses
Currey 2011 [75]	Australia	NA	NA	Program description	Clinical, clinical nurse research consultant
Currie 2013 [76]	United Kingdom	Qualitative	Descriptive	Cross-sectional	Network: Collaborations for Leadership in Applied Health Research and Care (CLAHRCs), senior managers, directors, academics, healthcare practitioners
Dawson 1998 [77]	Canada	NA	NA	Program description	Long term care facilities, none specific
Denis 2003 [78]	Canada	Quantitative	Descriptive	Cross-sectional	Network: Social research council, researchers and practitioners
Devon 2013 [79]	United States	NA	NA	Program description	Emergency department, physicians, postdoctoral nurses, principal investigators
Dickinson 2017 [80]	Australia	NA	NA	Commentary	None specific
Downie 2001 [81]	Australia	NA	NA	Program description	Hospital and university, nursing
Dupin 2014 [82]	France	Qualitative	Descriptive	Cross-sectional	Teaching hospitals, nurses
Ekeroma 2015 [83]	Pacific Islands	Quantitative	Intervention	Pre-post non-controlled study	Network: clinicians and stakeholders, obstetricians, gynaecologists, physicians, nurses, midwives
Ekeroma 2016 [84]	New Zealand	Quantitative	Measure	Cross-sectional	Reproductive health services in Pacific countries, nurses and doctors
Embi 2013 [85]	United States	NA	NA	Program description (proposed paradigm)	Healthcare broadly, none specific
Engler 2014 [86]	United states	NA	NA	Program description	National Institute of Nursing/Doctoral nursing programs, nurse doctoral students
Farmer 2002 [87]	Australia	NA	NA	Program description	Primary healthcare, GPs and primary healthcare practitioners
Fetherstonhaugh 2008 [88]	Australia	NA	NA	Program description	Sub-acute healthcare facility, nurse students
Finch 2013 [89]	Australia	Quantitative	Descriptive	Cross-sectional	Public healthcare workforce, speech pathologist
Fitzgerald 2003 [90]	United states	NA	NA	Program description (description of study)	Paediatric intensive care units, family of patients and staff nurse
Friesen 2014 [91]	Australia	NA	NA	Cross-sectional	Primary and community health services, hospital clinicians
Gagliardi 2009 [92]	Canada	Qualitative	Descriptive	Protocol	Faculties of nursing and medicine, physician and non-physician health service researchers
Gagliardi 2016 [93]	Canada	Qualitative	Descriptive	Cross-sectional	Healthcare delivery and healthcare monitoring organisation, researchers, clinicians, managers
Giles 2006 [94]	Australia	NA	NA	Commentary	The Centre of Clinical Research Excellence in Aboriginal and Torres Strait Islander Health / Aboriginal health Services, Indigenous people and communities
Gillibrand 2002 [95]	United Kingdom	NA	Descriptive	Program description	Clinical Networks for nursing research, nurse practitioners
Govoni 1997 [96]	United States	NA	NA	Case study	Clinical settings, clinical nurse specialists and staff nurses
Happell 2004 [97]	Australia	NA	NA	Program description	Psychiatric/mental health field, psychiatric nurses
Happell 2005 [98]	Australia	NA	NA	Commentary	Hospitals, nurses
Harrison 2005 [99]	United Kingdom	Qualitative	Descriptive	Cross-sectional	Primary care trusts (PCT), staff working directly or in partnership, GPs, primary care nurses on contract to PCT
Hauck 2015 [100]	Australia	Mixed Methods	Descriptive	Cross-sectional	Hospital, graduate midwives
Heinemann 2005 [101]	United States	NA	NA	Program description	Federal agencies, none specific
Heintzman 2014 [102]	United States	NA	NA	Commentary	Healthcare settings/practice based research networks, community practitioners
Henderson 2009 [103]	Australia	NA	NA	Commentary	Complex tertiary referral hospitals, nurse leaders
Hinchcliff 2014 [104]	Australia	NA	NA	Commentary	Health services more broadly, none specific
Hiscock 2014 [105]	Australia	Quantitative	Descriptive	Cross-sectional	Hospitals, doctors, nurses, allied health professionals
Hoeijmakers 2013 [106]	The Netherlands	Mixed Methods	Intervention	Prospective cohort	Academic Collaboration Centres for Public Health, students, science practitioners, management
Holden 2012 [107	Australia	Quantitative	Intervention	Non-randomised matched-pair trial	Primary health care, multidisciplinary primary healthcare teams
Holden 2012 [108]	Australia	Quantitative	Measure	Prospective	Queensland health, allied health workers
Holge-Hazelton 2016 [109]	Denmark	Qualitative	Descriptive	Cross-sectional	Regional hospital, advanced nurse practitioners
Hopps 1994 [110]	United Kingdom	NA	NA	Commentary	Nursing broadly, nurses
Hulcombe 2014 [111]	Australia	NA	NA	Program description	Health services/public sector health services broadly, health practitioners broadly
Hurst 2003 [112]	United Kingdom	Mixed Methods	Descriptive	cross-sectional	Health organisations, healthcare professionals
Ishiguro 2017 [113]	Japan	Quantitative	Descriptive	Cross-sectional	Children’s hospital, representative from hospitals
Jackson 2007 [114]	United Kingdom	NA	NA	Commentary	Research networks, nurses in general
Jackson 2015 [115]	Australia, UK	NA	NA	Commentary	Network, academics, consumers, clinicians, service organisations
Jamerson 2011 [116]	USA	NA	NA	Program description	Area nursing schools, nursing students
Jamerson 2012 [117]	United States	Quantitative	Descriptive	Cross-sectional	Network: nursing research society, nurse research facilitators
Janssen 2013 [118]	NZ	Mixed Methods	Intervention	Cross-sectional	Physio department of rehab hospital, physical therapists
Jennings 2013 [119]	Australia	NA	NA	Commentary	None specific
Johnson 2014 [120]	Australia	Quantitative	Descriptive	Cross-sectional	Cancer services, health professionals
Jordan 2015 [121]	United Kingdom	Qualitative	Descriptive	Cross-sectional	National Institute for Health CLAHRC, members of CLAHRC
Joubert 2015 [122]	Australia	NA	NA	Commentary	Oncology social work department, social workers
Jowett 2000 [123]	United Kingdom	Quantitative	Descriptive	Cross-sectional	Primary care, GPs in service practice
Keefe 1998 [124]	United States	NA	NA	Program description	Denver Collaborative Research Network, nurses
Keenan 2000 [125]	Canada	NA	NA	Commentary	Critical care research network/healthcare broadly, healthcare workers
Koerner 2014 [126]	United States	NA	NA	Program description	Network: collaborative projects with practitioners and researchers, clinicians and researchers
Kuehnle 2009 [127]	Switzerland	Quantitative	Descriptive	Cross-sectional	Swiss national MD-PhD program, former and current students
Lach 1980 [128]	United States	NA	NA	Commentary	College health, students
Ledley 1993 [129]	United States	Quantitative	Descriptive	Cross-sectional	Children’s hospital, Paediatrician residents
Legris 2000 [130]	Canada	Quantitative	Descriptive	Retrospective study	Clinical in-patient units, Multidisciplinary practitioners
Lester 1998 [131]	United Kingdom	Quantitative	Descriptive	Cross-sectional	UK departments of general practice, practitioners
Long 2014 [132]	Australia	Mixed Methods	Descriptive	Cross-sectional	Translational research networks (TRNs), members
Loomis 1980 [133]	USA	NA	NA	Program description	Network: collaborative research program, nurse researchers and nurse clinicians
MacPhee 2009 [134]	Canada	NA	NA	Program description (describing a model)	Large urban community hospital and nursing facility of a local university, first line nurse leaders and educators
Mainous 1995 [135]	United States	Quantitative	Descriptive	Cross-sectional	General or family practice, general internal medicine or paediatrics, primary care physicians
Mays 2013 [136]	United States	Quantitative	Descriptive	Cross-sectional	Practice based research networks, representatives of public health organisations
McAleavey 2015 [137]	United States	NA	NA	Commentary	University and college counselling centres, counsellors, administrators and researchers
McIntyre 2011 [138]	Australia	Mixed Methods	Descriptive	Cross-sectional	Academic departments of general practice and rural health
McKee 2017 [139]	Ireland	Mixed Methods	Intervention	Cross-sectional (post intervention only)	Acute urban hospital, advanced nursing staff, nurse researchers and academic partners
McWilliam 1997 [140]	Canada	NA	NA	Program description	Network: research partnership
Miller 2009 [141]	Canada	NA	NA	Program description	Health Authority, organisation members of health
Misso 2016 [142]	Australia	Mixed Methods	Intervention	Protocol	Clinical setting, health professionals
Mitchell 2015 [143]	United Kingdom	NA	NA	Commentary	University College London Hospitals, nurses and midwives
Moore 1997 [144]	United Kingdom	NA	NA	Commentary	Clinical settings, physiotherapists
Morris 2017 [145]	United Kingdom	Mixed Methods	Descriptive	Cross-sectional	Mental health services, occupational therapists
Mortenius 2015 [146]	Sweden	Qualitative	Descriptive	Prospective	Child care centre, primary care staff
Murphy 2015 [147]	United States	NA	NA	Commentary	Healthcare broadly, doctor of nursing practice-prepared and doctor of nursing philosophy-prepared nurses, students and faculty
Naik 2015 [148]	United States	Qualitative	Intervention	Cross-sectional	Primary care settings, health services research team, primary care community and US VA health system
Nelson 2007 [149]	United States	NA	NA	Commentary	Clinical practice settings, clinical nurse specialist
Nichols 1997 [150]	United Kingdom	NA	NA	Commentary	Healthcare settings, none specific
Norton 2011 [151]	England	Qualitative	Descriptive	Cross-sectional	Local Research Network, chief pharmacists
Norman 1987 [152]	United States	NA	NA	Commentary	Nurses
Nutting 1996 [153]	United States	NA	NA	Commentary	Primary care practice/practice based research networks, family physicians
Pager 2012 [154]	Australia	Qualitative	Descriptive	Cross-sectional	Queensland Health, allied health professionals
Paget 2014 [155]	Australia	Quantitative	Descriptive	Cross-sectional	Tertiary children’s hospital, doctors, nurses and allied health professionals
Paget 2017 [156]	Australia	Qualitative	Descriptive	Cross-sectional	Tertiary children’s hospital, paediatric clinicians
Perron 2016 [157]	Canada	Quantitative	Intervention	Post intervention assessment	Rehabilitation research network, paramedic clinicians
Pikethly 2003 [158]	United Kingdom	NA	NA	Program description	Primary care research and development network/primary care, clinical staff
Pomeroy 2003 [159]	United Kingdom	Mixed Methods	Intervention	Cross-sectional	Stroke association therapy network unit, clinicians
Queensland Health 2016 [160]	Australia	Qualitative	Descriptive	Cross-sectional	Queensland Health sites, clinicians, team leaders, professional heads
Rait 2002 [161]	United Kingdom	Mixed Methods	Descriptive	Cross-sectional	NHS organisations, NHS staff
Reay 2013 [162]	United Kingdom	NA	NA	Program description (model)	None specific
Redman-MacLaren 2010 [163]	Soloman Islands (reporting)/Australia (written)	Mixed Methods	Intervention	Cross-sectional	Adventist hospital, health professionals
Reijneveld 2009 [164]	Netherlands	NA	NA	Commentary	Public health practice
Rickels 1977 [165]	United States	NA	NA	Commentary	Family practice, private practice research group
Ried 2005 [166]	Australia	NA	NA	Commentary	Primary health care, practitioners
Rosenberg 1999 [167]	United States	NA	NA	Commentary	Physician scientists
Sahs 2016 [168]	United States	Qualitative	Descriptive	Cross-sectional	Hospital based outpatient clinic, clinicians and administrators
Schwartz 1987 [169]	England	NA	NA	Commentary	None specific, academic pharmacists
Short 2010 [170]	Australia	NA	NA	Commentary	Clinical setting broadly, emergency department
Short 2009 [171]	Australia	Mixed Methods	Descriptive	Cross-sectional	Hospital, doctors, nurses and allied health professionals
Skinner 2014 [172]	Australia	NA	NA	Commentary	Tertiary hospital, physiotherapists
Smolowitz 1997 [173]	United States	Quantitative	Descriptive	Cross-sectional	Acute care facilities, nursing schools, staff
Soper 2015 [174]	United Kingdom	Mixed Methods	Intervention	Prospective cohort	Partnership between universities and local NHS organisations, none specific
Stiller 2016 [175]	Australia	Quantitative	Descriptive	Cross sectional	NA
Stockton 2010 [176]	USA	NA	NA	Commentary	Clinical, psychotherapists
Tanner 2002 [177]	United Kingdom	Mixed Methods	Descriptive	Cross-sectional	NHS Trust, research active clinical nurses
Teal 2012 [178]	United States	Qualitative	Measure	Cross-sectional	Community practice, physicians, administration and support staff
The Academy of Medical Sciences [179]	United States	NA	NA	Commentary	None specific
Tierney 1991 [180]	Scotland	Mixed Methods	Descriptive	Prospective cohort	Nurses and University Nursing Research Unit, nurses
Walshe 2013 [181]	England	NA	NA	Commentary	Health research broadly, none specific
Willson 2010 [182]	USA	NA	NA	Program description	Network: Collaborative Paediatric Critical Care Research Network, physicians and researchers
Wolfenden 2017 [183]	Australia	NA	NA	Program description	Population Health, researchers and health service staff
Woolf 2008 [184]	USA	NA	NA	Commentary	NA
Yawn 2002 [185]	United States	NA	NA	Commentary	Family medicine

Of the databased articles (*n* = 68), 17 (25%) studies were mixed methods, 23 (34%) were qualitative and 28 (41%) were quantitative. The majority (*n* = 49, 72%) were categorised as descriptive studies, 17 were intervention studies (25%) and two were measurement studies (3%). Of the non-databased articles (*n* = 84), the majority (*n* = 46, 55%) were commentaries, one was a case study (1%) and the remainder were program descriptions (*n* = 37, 44%).

ii. **Study design**

Of the 18 data based studies that were interventions, only two were controlled studies [74, 107] and one was a protocol of a controlled trial [142]. Nine studies were post-intervention, cross sectional assessments without control arms [34–42], five were prospective or retrospective cohorts, pre-post studies, or repeat cross sectional assessments [54–58].

iii. **Study setting and participants**

Of the 68 databased articles, 29 studies were conducted either in hospital(s) or a department within a hospital, seven were in health care settings more broadly, four were in community health care settings, 16 explored networks (usually a collaboration of academic/health care partnerships and institutions), 11 were in primary care, and one in a university. There were a range of participants in these studies including Aboriginal Health Workers, nurses, midwives, physiotherapists, primary care practitioners, pharmacists, allied health practitioners, community health care workers and practitioners, clinicians and administrators within particular health systems or context (i.e. NHS, CLARHC), counsellors, ICU staff, multidisciplinary primary health care team and medical students.

### Qualitative synthesis of strategies

Table 3 outlines the strategies identified in all studies that described an intervention (both databased and non-databased) and examples of suggested/actual application in practice. We identified and described eight categories of strategies: dual skilled team/staff, resources or physical infrastructures, incentive, leadership support of research, education/training, networks, forming partnerships or collaborations and overall leadership for entity.

**Table 3:** Qualitative synthesis of research engagement strategies outlined in all included studies

Research engagement strategy	Definition	Examples	n*
Research trained/skill staff within health care settings	Including research-trained staff embedded/integrated within practice settings. Staff may be located physically and/or included into service delivery teams and /or organisational governance positions to facilitate enhanced research and service delivery	Research-practice roles (i.e. nurse researcher; clinical nurse research consultant; physician-scientist; lecturer practitioner); creating new positions i.e. positions for research fellows; executive nursing positions, financial support (salaries); funded/guaranteed research time; secondments ( i.e. a joint position hired by practice and university, co-location of staff exchange of staff ( i.e. clinical teaching to the students in exchange for research support and clinical staff’s opportunities for continuing education), fellowships/positions to undertake research activity as part of their role (e.g. Service Delivery Management Fellowship programme [59]	3
Resources/infrastructure	Provision of resources dedicated for research activity. This includes the availability of specific tools, funding, administrative support and mentoring specifically allocated to research activity	Provision of print/online journal subscription; available research space (desk, computer, software); information technology support; stats support; facilities; equipment; maintenance; research support centre; data collection tools; data infrastructure; admin support; on-site research supervisors; support research staff; research coordinators, ethics application support, ethics application process streamlined across partner sites; standard operating procedures where research can be bedded into practice; common quality assurance mechanisms to support evaluation; organisational support in collaborative development of research proposals, funding for dissemination (conferences, open access publications); access to local funding for ongoing use	90
Organisational incentive and rewards	Financial or non-financial rewards for undertaking research or non-tangible incentives such as increase in stature. This strategy seeks to recognise formally clinician’s time needed to undertake research activity and puts in place structures that enable this	Reimbursements for undertaking research; credits toward professional licenses; scholarship and paid research placements, dedicated research grants, bursaries; PhD tuition support and scholarships, awards, honours; public recognition of research excellence, flexible working arrangements/job descriptions (re-adjusted per research project needs); flexible salaries for PhD training	10
Leadership supportive of research	Commitment, involvement, accountability of leaders and managers to support research. This includes strategies to ensure leadership buy in and support for research activity	Leadership formally endorsing research (i.e. this includes a strategic plan that reflects a strong commitment to developing research activity and research capacity), leadership in building research questioning and uncertainty into clinical service’s culture. Managerial support by releasing practitioner time to undertake research	62
Education/training/capacity building	This includes providing research capacity building opportunities and specifically targets the knowledge, skills and self-efficacy of practitioners to undertake research	Provision of a range of training and research capacity building activities including online training; seminars; workshop; professional development, one-on-one advice/meetings; role modelling; local champions/behavioural coaches; simulation; experiential learning; mentoring (including, peer mentoring and senior staff/expert mentoring); supervision; teaching opportunities; research forum and conferences lecture series; journal clubs Support to obtain formal qualifications/training Participatory Action Research cycle used for capacity building/engagement: Observe-Act-Plan-Reflect used to encourage practitioners to initiate and lead res projects in their areas of interest Supporting medical students to undertake research (mentoring, summer research fellowships, research portals)	93
Networks and communication	This strategy targets the nature and quality of social networks and the nature and quality of formal and informal communications. It recognises the research engagement is enhanced by academic collaborations and formal communication activities	Forums/conferences/multi-disciplinary workshops, curricula, meetings, seminars aimed at building relationship and collaboration, site inspections; joint PhD student supervision between practice and academic institutions, projects and technologies shared between collaborators; committees with institutional representation from a range of organisations, engagement and communication activities for research uptake with a range of end-user organisations	7
Partnership/collaborations between practice and academic institutions	The extent of partnership and collaboration with academic institutions. It moves beyond consultant-based approaches to formal joint research collaboration and activities	Leveraging material and in-kind resources to achieve shared goals; joint plans for implementation of projects; shared governance and leadership of research projects (i.e. executive committee with membership from all organisations, co-chaired steering committee; board of governors; collaborative agreements; formal written contract signed between institutions)	12
A formal research-practice entity that is government funded	These are established formal structures in which roles formalised to enhance partnerships between universities and health care systems to facilitate clinician-led research	Examples include Collaborations for Leadership in Applied Health Research and Care (CLARHC) in the UK ($88 million pound commitment over five years with the requirement that each CLARCH match the funding from local NHCS organisations)	3

^*^n equals more than 100 as some studies described multiple strategies

## Discussion

This scoping review sought to provide an overview of the existing research exploring strategies that have been suggested to improve provider and health care organisation’s engagement with research. This review found that over half of publications describing research engagement did not formally present any new data (non-databased and opinion pieces). Of the databased publications (those that formally presented new data), the majority were descriptive in nature largely providing evidence of the extent of the behaviour and association between strategies and research engagement. Our study found only two controlled trials evaluating the impact of research engagement strategies [74, 107]. Similarly, the review by Gagliardi et al. in 2015, also found only 13 studies, none were controlled studies, describing how iKT approaches have been operationalised and used to improve collaboration between researchers and decision makers in the research process [15]. Such findings are consistent with that of an emerging field, however, are of concern given significant resource investment by governments internationally to establish structures and implement strategies to build research leadership and capacity in health care organisations. For example, the NIH Roadmap allocated an initial $125 million in 2004, with planned increases to $2 billion in the later stages of implementation [186] where funds are distributed across developing new innovations, developing new models for research and increasing research translation.

Encouragingly, the percentage of databased and intervention studies appears to be increasing over time although the overall number is still small. Using a FA, we identified and described strategies that were suggested to be useful, or had been previously applied to increase clinician and health care organisations engagement with research, with the intent of providing a list of strategies that could be applied and evaluated in future studies. The research engagement strategies were described across eight categories targeting clinician, team, organisation and supra organisation factors [71]. Such strategies seek to target the broader determinant described by Gagliardi et al. and are consistent with that outlined in the research co-production literature [187], which suggests that interventions need to move beyond targeting just knowledge and skills of clinicians. Findings from this review also add to research capacity building frameworks for practitioners by describing the broader collaborative (e.g. networks), structural or workforce arrangements (e.g. clinician researchers) that could be put in place to support clinical research leadership [16]. Further intervention research is warranted targeting these eight distinct categories to better understand the impact of such strategies on increasing clinician research engagement. Additionally, a systematic review of the descriptive literature may also provide additional insights into the association of such strategies with engagement outcomes.

Our review highlights a mismatch between investment in research engagement strategies and the available evidence to support such strategies. While initiatives like the NIHR CLARHC provided significant opportunity and funding to embed a broad range of strategies to facilitate research co-production and clinician-led research, the impact these structures have on research engagement have yet to be examined [188]. Further, to our knowledge, there have been no controlled evaluations of multi-level formalised programs such as CLAHRC [188]. This may be due to the challenges with defining important outcomes of multi-level initiatives that account for the individualised approaches taken by different sites to contextualise the intervention. These non-controlled evaluations provide rich and important data to understand the factors [189] that facilitate implementation and the context in which these initiatives are delivered. However evidence from these non-controlled evaluations needs to be coupled with rigorous comparative evaluations to provide essential evidence for decision-making and to justify continued investments.

### Strengths/limitation

This study used high quality, systematic processes to ensure that a broad range of studies examining research engagement were included. At least two screeners were included in each of the review processes. The use of structured qualitative processes to generate a list of strategies allowed for a broad examination of the types of strategies previously discussed or applied in research and practice, and enabled a collaborative analyses by researchers with varying degrees of qualitative research experience.

While a comprehensive search strategy consisting of a database search, search of the grey literature and consultations with experts in the field was used, it is possible that studies were missed due to inconsistent terminology and the rapidly progressing evidence-base. We excluded non-English studies and as such could have missed potentially relevant studies published in other languages. Nevertheless, this study to our knowledge provides a comprehensive overview of the characteristics of studies examining research engagement using high quality processes. This scoping review highlights that future empirical research is needed to identify the impact of such proposed strategies on health services, practice and patient outcomes.

## Conclusions

This scoping review for the first time provides a list of research engagement strategies that have been proposed to be useful to increase clinicians and health care organisations collaboration in research activity. The majority of the evidence however has been descriptive in nature, providing limited empirical evidence to support the efficacy of research engagement strategies. There is a need for future research to progress beyond descriptive research to methodologically rigorous intervention research, to provide the evidence needed to inform decision making.

## Supplementary Information

**Additional file 1.** Electronic search strategy applied for the search.

**Additional file 2.** Data extraction form.

## Data Availability

The datasets used and/or analysed during the current study available from the corresponding author on reasonable request.
