# Supplementary material for: Describing the evidence-base for research engagement by health care providers and health care organisations: a scoping review
Source: BMC Health Serv Res. 2023 Jan 24;23:75. doi: 10.1186/s12913-022-08887-2 (PMC9872336; doi:10.1186/s12913-022-08887-2)
Supplement: Supplementary file 1 — Additional file 1. Electronic search strategy applied for the search. [file 12913_2022_8887_MOESM1_ESM.docx]

Additional File 1: Electronic Search Terms.

Database(s): **Ovid MEDLINE(R) Epub Ahead of Print, In-Process & Other Non-Indexed Citations, Ovid MEDLINE(R) Daily and Ovid MEDLINE(R)**1946 to Present 
Search Strategy:

| **#** | **Searches** |
| --- | --- |
| 1 | health services research/ |
| 2 | translational medical research/ |
| 3 | organizational culture/ |
| 4 | organizational innovation/ |
| 5 | (research* adj2 (translation* or network* or engag* or collaborat*)).mp. |
| 6 | 1 or 2 or 3 or 4 or 5 |
| 7 | clinical nursing research/ |
| 8 | Allied Health Personnel/st, sn [Standards, Statistics & Numerical Data] |
| 9 | Physicians/st, sn [Standards, Statistics & Numerical Data] |
| 10 | Nurse Clinicians/st, sn [Standards, Statistics & Numerical Data] |
| 11 | Research Personnel/st, sn [Standards, Statistics & Numerical Data] |
| 12 | Hospitals/ |
| 13 | Health Services/ |
| 14 | acute care hospital*.mp. |
| 15 | clinical setting*.mp. |
| 16 | 7 or 8 or 9 or 10 or 11 or 12 or 13 or 14 or 15 |
| **17** | **6 and 16** |

Database(s): **Embase**1947 to present 
Search Strategy:

| **#** | **Searches** |
| --- | --- |
| 1 | health services research/ |
| 2 | translational research/ |
| 3 | (organi?ational adj (culture or innovation)).mp. [mp=title, abstract, heading word, drug trade name, original title, device manufacturer, drug manufacturer, device trade name, keyword, floating subheading word] |
| 4 | (research* adj2 (translation* or network* or engag* or collaborat*)).mp. |
| 5 | 1 or 2 or 3 or 4 |
| 6 | clinical nursing research/ |
| 7 | *paramedical personnel/ and (standard* or statistic* or numerical data).tw. |
| 8 | *physician/ and (standard* or statistic* or numerical data).tw. |
| 9 | *clinical nurse specialist/ and (standard* or statistic* or numerical data).tw. |
| 10 | Research Personnel.mp. |
| 11 | *hospital/ |
| 12 | *health service/ |
| 13 | acute care hospital*.mp. |
| 14 | clinical setting*.mp. |
| 15 | 6 or 7 or 8 or 9 or 10 or 11 or 12 or 13 or 14 |
| **16** | **5 and 15** |

Database(s): **PsycINFO**1806 to November Week 2 2017 
Search Strategy:

| **#** | **Searches** |
| --- | --- |
| 1 | organizational climate/ |
| 2 | (organi?ational adj (culture or innovation)).mp. |
| 3 | (research* adj2 (translation* or network* or engag* or collaborat* or health service*)).mp. |
| 4 | 1 or 2 or 3 |
| 5 | exp Allied Health Personnel/ |
| 6 | exp PHYSICIANS/ |
| 7 | exp Nurses/ |
| 8 | Research Personnel.mp. |
| 9 | exp HOSPITALS/ |
| 10 | Health Services.mp. |
| 11 | acute care hospital*.mp. |
| 12 | clinical setting*.mp. |
| 13 | 5 or 6 or 7 or 8 or 9 or 10 or 11 or 12 |
| **14** | **4 and 13** |

CINAHL

| **#** | **Query** |
| --- | --- |
| S1 | (MH "Health Services Research") |
| S2 | (MH "Organizational Culture") |
| S3 | "organi?ational innovation" |
| S4 | (research* n2 (translation* or network* or engag* or collaborat*)) |
| S5 | S1 OR S2 OR S3 OR S4 |
| S6 | (MH "Clinical Nursing Research") |
| S7 | (MH "Allied Health Personnel/SN/ST") |
| S8 | (MH "Physicians/SN/ST") |
| S9 | (MH "Clinical Nurse Specialists/ST/SN") |
| S10 | (MH "Research Personnel/ST/SN") |
| S11 | (MH "Hospitals") |
| S12 | (MH "Health Services") |
| S13 | acute care hospital* |
| S14 | "clinical setting*" |
| S15 | S6 OR S7 OR S8 OR S9 OR S10 OR S11 OR S12 OR S13 OR S14 |
| **S16** | **S5 AND S15** |

**Academic Search Ultimate**

| **#** | **Query** |
| --- | --- |
| S1 | TI "health services research" OR AB "health services research" |
| S2 | TI "translational medical research" OR AB "translational medical research" |
| S3 | TI "organi?ational culture" OR AB "organi?ational culture" |
| S4 | TI "organi?ational innovation" OR AB "organi?ational innovation" |
| S5 | TI ( (research* n2 (translation* or network* or engag* or collaborat*)) ) OR AB ( (research* n2 (translation* or network* or engag* or collaborat*)) ) |
| S6 | S1 OR S2 OR S3 OR S4 OR S5 |
| S7 | TI "clinical nursing research" OR AB "clinical nursing research" |
| S8 | TI "Allied Health Personnel" OR AB "Allied Health Personnel" |
| S9 | TI Physicians OR SU Physicians |
| S10 | TI "Nurse Clinician*" OR AB "Nurse Clinician*" |
| S11 | TI "Research Personnel" OR AB "Research Personnel" |
| S12 | TI Hospital* OR AB Hospital* |
| S13 | TI "Health Services" OR AB "Health Services" |
| S14 | TI "clinical setting*" OR AB "clinical setting*" |
| S15 | S7 OR S8 OR S9 OR S10 OR S11 OR S12 OR S13 OR S14 |
| **S16** | **S6 AND S15** |
