# Supplementary material for: Describing the evidence-base for research engagement by health care providers and health care organisations: a scoping review
Source: BMC Health Serv Res. 2023 Jan 24;23:75. doi: 10.1186/s12913-022-08887-2 (PMC9872336; doi:10.1186/s12913-022-08887-2)
Supplement: Supplementary file 2 — Additional file 2. Data extraction form. [file 12913_2022_8887_MOESM2_ESM.docx]

**Additional file 2: Data extraction form**

|  | **Type of information extracted** |
| --- | --- |
| Source the publication was obtained from | Grey literature, expert information or databased search |
| Author, Year | First author name, and year of publication |
| Country | Country where research was undertaken |
| Databased/non-databased | Whether the study reported new data or no new data |
| Study design | As described by the study, and qualitative or quantitative as described by the study or using analysis where not described |
| Setting | Primary setting where the research was undertaken |
| Participants | Information regarding participants |
| Terms to describe research engagement | Extracted verbatim from text |
| Intervention details | Extracted verbatim from text |
| Outcomes | Types of outcomes assessed |
